# Trend in geographic distribution of physicians in Japan

**DOI:** 10.1186/1475-9276-8-5

**Published:** 2009-03-03

**Authors:** Shin-ichi Toyabe

**Affiliations:** 1 Crisis Management Office, Niigata University Medical and Dental Hospital, Asahimachi-Dori1-754, Chuo-Ku, Niigata City 951-8520, Japan

## Abstract

**Background:**

Since the late 1980s, the policy of the Japanese government regarding physician manpower has been to decrease the number of medical students. However, the shortage of doctors in Japan has become a social problem in recent years. The aim of this study was to compare the numbers of physicians in Japan between 1996 and 2006 and the trends in distribution of physicians.

**Methods:**

The time trends in number and distribution of physicians between 1996 and 2006 were analyzed. Gini coefficient, Atkinson index and Theil index were used as measures for mal-distribution of physicians to population. The distribution of physicians was visualized on a map by using geographic information system (GIS) software.

**Results:**

The total number of physicians increased every year in the period from 1996 to 2006 but has remained below the international standard. All three measures of mal-distribution of physicians worsened after 2004, and the worsening was remarkable in the distribution of physicians working at hospitals. The number of physicians working at hospitals has significantly increased in urban areas but not in areas with low population densities. When medical interns were excluded from calculation, the measures of mal-distribution improved.

**Conclusion:**

The problem of a doctor shortage in Japan is linked to both the shortage of absolute number of physicians and the mal-distribution of hospital physicians. The new postgraduate internship system might worsen this situation.

## Background

Since the late 1980's, the policy of Japanese government regarding physicians' manpower has been to decrease the number of medical students because of the predicted surplus of doctors. Student quotas for medical schools were decreased by 7.8% from 1986 to 2006. However, the shortage of doctors in Japan has recently become a serious social problem, which has been repeatedly highlighted in mass media [1,2].

The number of physicians in Japan is small compared with the numbers in other developed countries. Japan ranks 60th in terms of number of physicians per 1,000 population among WHO's 193 member states [3]. The number of physicians per 1,000 population in Japan was 1.98 in 2002, whereas it was 2.56 in the United States in 2000 and 2.30 in the United Kingdom in 1997. Japan belongs to the lowest group among Organization for Economic Cooperation and Development (OECD) countries, together with Mexico, South Korea and Turky. On the other hand, demand of physicians in Japan is greater than other countries. Healthcare utilization in Japan is high, and the number of consultations per capita is the highest among OECD countries [4]. High utilization of hospitals by patients in Japan has exposed the shortage of physicians.

Although there have been absolute and relative deficiencies in the number of physicians in Japan, it is not clear why the physician shortage problem has recently emerged as a significant social issue. This problem has often been discussed in relation to a new internship system for medical school graduates that was introduced in 2004 [5-7]. New graduates from medical schools now have to complete two years of internship at hospitals before they start professional carriers. This policy change might affect the supply and distribution of physicians in Japan [8]. However, there have been few reports on the number of physicians and the distribution of physicians in Japan. Kobayashi et al. Conducted a comparative study on the number and distribution of physicians in Japan in 1980 and 1990 [9]. They found that the inequality in physician distribution in Japan did not improve despite of an increase in the number of physicians from 1980 to 1990. However, the trends in number and distribution of physicians since 1990 are not known. The aim of this study was to compare the numbers of physicians in Japan between 1996 and 2006 and the trends in distribution of physicians.

## Methods

Japan consists of 47 prefectures, and each prefecture consists of many municipal bodies such as cities, towns and villages. There were 3,370 municipal bodies in 1996, but the number of municipal bodies decreased to 1,973 in 2006 due to municipal merger (Table 1). Physicians in Japan must inform to the Ministry of Health, Labour and Welfare of Japan (MHLW) every two years the place in which they work (clinic, general hospitals or university hospital). The MHLW has published data on the number of physicians working at each municipal body [10]. The data used for analyses in this study included data for six time periods spanning one decade: 1996, 1998, 2000, 2002, 2004 and 2006. Only data for practicing physicians were used in this study, and data for physicians who were basic researchers or government officers were excluded from analyses. Physicians were categorized into three groups according to institutions where they practiced: general hospitals, university hospitals and clinics. This categorization is reasonable since Japanese physicians working at hospitals do not have their own private clinics.

**Table 1:** Numbers of physicians and municipal bodies each year

Year	Number of practicing physicians	Population (×1,000)	Number of physicians per 100,000 population	Number of municipal bodies
1996	230,297	125,864	183.0	3,370
1998	236,933	126,486	187.3	3,371
2000	243,201	126,926	191.6	3,368
2002	249,574	127,435	195.8	3,359
2004	256,668	127,687	201.0	3,074
2006	263,540	127,770	206.3	1,973

Three measures of mal-distribution of physicians were calculated for each time period, fundamentally based on the ratio of physicians to population in each municipal body. The measures of mal-distribution were the Gini coefficient, Atkinson index and Theil index. These measures were initially designed to analyze inequality of income or wealth, and they have been used to study the distribution of health resources such as physician distribution [9,11-15]. Lower values of these measures indicate more equal distribution. For example, the Gini coefficient is between zero (perfect equality) and one (perfect inequality). All three measures were calculated by methods described elsewhere [9,11-15]. The Gini coefficient was calculated from the Lorenz curve and the coefficient ε was set at 0.5 to calculate the Atkinson index. Since the number of municipal bodies in Japan has been changing due to municipal mergers (Table 1) and these measures might be affected by the number of municipal bodies, boundaries of municipal bodies were reconstructed to the 2006 boundaries using geographic information system (GIS) software [16]. The three measures of mal-distribution were calculated using the reconstructed data.

The areas in which the number of physicians had increased or decreased in recent years were then analyzed in detail. Municipal bodies were categorized by the size of their population, and changes in physicians-to-population ratio in each category were analyzed. The physicians-to-population ratio is shown by median (25-percentile, 75-percentile).

Finally, the distribution of physicians was plotted onto 10-km mesh maps by using the GIS software in order to visualize the spatial distribution of geographic regions in which the number of physicians had increased or decreased.

All analyses except for spatial analyses were performed using SPSS 15.0J (SPSS Japan Inc., Tokyo, Japan). Spatial analyses and plotting on maps were performed by using MapCall Standard 2.1 (Chuo Group Inc., Niigata, Japan) and ArcGIS 9.2 (ESRI Japan Inc., Tokyo, Japan).

## Results

### Time trend in number of practicing physicians in Japan

**Figure 1:**
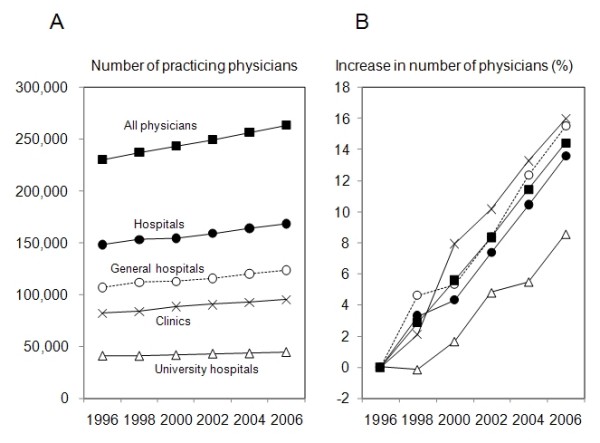
**Year-to-year trends in numbers of physicians in Japan**. Numbers of physicians practicing at general hospitals (open circles), university hospitals (open triangles) and clinics (crosses) in six time periods are shown (A). Increment ratios in numbers of physicians compared with those in 1996 are also shown (B).

Both the total number of physicians and the average ratio of physicians to population have been increasing every year (Figure 1, Table 1). The number of practicing physicians has been increasing by 3,000 (1.3%) every year. At present, there are about 260,000 practicing physicians in Japan, and the overall ratio of practicing physicians to 100,000 population is 206.3 (Table 1). Forty-seven percent of physicians are working at general hospitals, 36% are practicing at clinics, and 17% are working at university hospitals.

### Year-to-year trends in measures of mal-distribution

**Figure 2:**
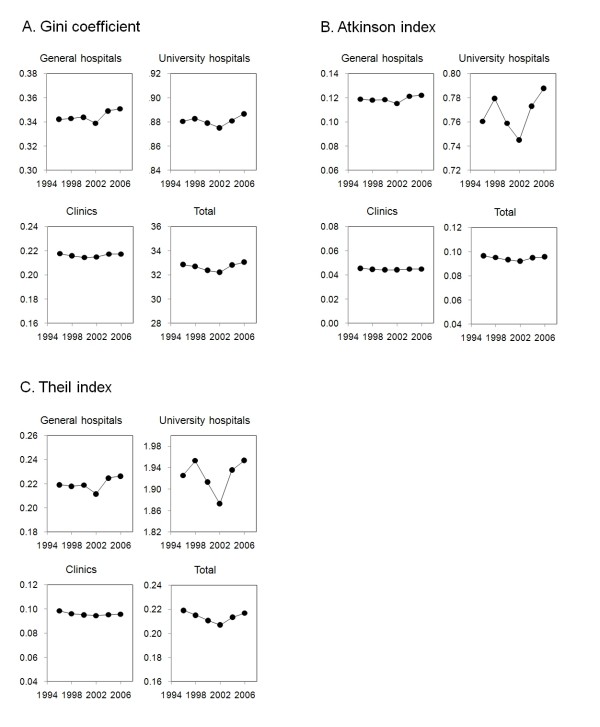
**Year-to-year trends in Gini coefficient, Atkinson index and Theil index for distribution of physicians**. Measures of mal-distribution for physicians practicing at hospitals, general hospitals and clinics and total number of physicians in six time periods between 1996 and 2006 are shown.

The three measures of mal-distribution showed similar trends from 1996 to 2006 (Figure 2). They remained at approximately the same level or improved slightly until 2002. The turning point was 2004, when all of the three measures for distributions of physicians working at general hospitals and physicians working at university hospitals deteriorated. The measures remained high in 2006. On the other hand, the three measures of mal-distribution for physicians working at clinics remained at almost the same level from 1996 to 2006.

### Changes in the distribution of physicians during 2004

**Figure 3:**
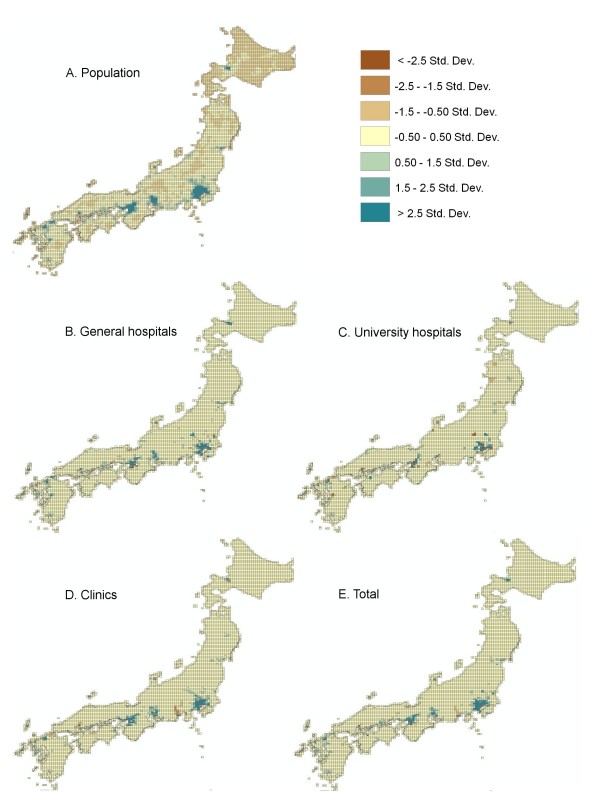
**Changes in number of physicians from 2002 to 2006**. Differences in numbers of physicians between 2002 and 2006 were plotted on 10-km mesh maps. Distribution of population (A) and increases in number of physicians working at general hospitals (B), number of physicians practicing at university hospitals (C), number of physicians practicing at their own clinics (D) and total number of physicians are shown.

Since the measures of mal-distribution worsened after 2004, we analyzed in more detail the areas in which the number of physicians had increased or decreased. We categorized municipal bodies by size of their population and analyzed changes in ratio of physicians to 100,000 population around 2004 in each category of municipal bodies (Table 2). Concentration of physicians in larger urban areas was observed for all three categories of physicians, but the tendency was most remarkable in the case of physicians working at general hospitals. Numbers of physicians working at general hospitals were larger in areas with a large population than in areas with a small population. Increase in the ratio of physicians to 100,000 population from 2002 to 2006 was also higher in areas with high population density than in areas with low population density. This tendency was not so obvious in the case of physicians working at clinics. To analyze in detail the distribution of physicians and its time trend, we plotted numbers of physicians in 2002 and 2006 and their difference onto 10-km mesh maps (Figure 3). Physicians were distributed unequally in Japan, and increase in the number of physicians working at general hospitals was prominent in urban areas with large populations such as Tokyo, Nagoya and Osaka (Figure 3A and Figure 3B). The number of physicians working at university hospitals increased mainly in the Tokyo metropolitan area (Figure 3C). On the other hand, the number of physicians working at hospitals remained unchanged in rural areas and decreased in areas surrounding large cities.

**Table 2:** Changes in the ratio of physicians per 100,000 population from 2002 to 2006

Size of population of municipal bodies	n	Median number of physicians in each municipal body			Change in median between 2002 and 2006
			
		2002	2004	2006	
*Physicians working at general hospitals*					
<5,000	233	0.0 (0.0, 0.0)	0.0 (0.0, 0.0)	0.0 (0.0, 0.0)	0.0
5,000–10,000	273	0.0 (0.0, 51.4)	0.0 (0.0, 51.1)	0.0 (0.0, 61.5)	0.0
10,000–30,000	509	39.1 (12.1, 79.5)	38.9 (10.6,77.9)	44.0 (13.1, 81.5)	4.9
30,000–50,000	269	60.7 (30.9, 99.1)	61.2 (32.2, 99.1)	66.2 (34.6, 108.6)	5.5
50,000–100,000	301	69.1 (43.9, 102.9)	68.5 (52.7,120.7)	76.2 (49.3, 106.6)	7.1
100,000–300,000	317	81.8 (50.3, 115.2)	86.3 (52.7, 120.7)	95.3 (60.9, 132.9)	13.5
≥300,000	71	77.7 (51.2, 105.1)	81.5 (52.7, 117.1)	101.0 (73.1, 134.1)	23.3
					
*Physicians working at university hospitals*					
<5,000	233	0.0 (0.0, 0.0)	0.0 (0.0, 0.0)	0.0 (0.0, 0.0)	0.0
5,000–10,000	273	0.0 (0.0, 0.0)	0.0 (0.0, 0.0)	0.0 (0.0, 0.0)	0.0
10,000–30,000	509	0.0 (0.0, 0.0)	0.0 (0.0, 0.0)	0.0 (0.0, 0.0)	0.0
30,000–50,000	269	0.0 (0.0, 0.0)	0.0 (0.0, 0.0)	0.0 (0.0, 0.0)	0.0
50,000–100,000	301	0.0 (0.0, 0.0)	0.0 (0.0, 0.0)	0.0 (0.0, 0.0)	0.0
100,000–300,000	317	0.0 (0.0, 2.6)	0.0 (0.0, 0.0)	0.0 (0.0, 1.6)	0.0
≥300,000	71	2.0 (0.3, 76.4)	2.1 (0.0, 66.1)	4.3 (0.4, 92.9)	2.3
					
*Physicians working at clinics*					
<5,000	233	41.5 (20.5, 66.3)	39.1 (20.4, 63.1)	45.2 (23.9, 75.7)	3.7
5,000–10,000	273	34.4 (22.4, 50.0)	33.1 (18.9, 48.5)	37.0 (21.7, 55.0)	2.6
10,000–30,000	509	41.5 (27.3, 58.8)	41.3 (27.9, 57.6)	45.7 (31.8, 61.1)	4.2
30,000–50,000	269	54.0 (39.7, 69.1)	53.4 (38.5, 70.0)	56.9 (45.2, 74.0)	3.0
50,000–100,000	301	58.1 (44.1, 70.8)	57.9 (44.8, 75.6)	63.3 (52.1, 79.5)	5.2
100,000–300,000	317	64.9 (49.7, 80.2)	65.8 (52.8, 83.7)	70.5 (57.4, 89.4)	5.6
≥300,000	71	72.4 (49.9, 84.5)	75.7 (53.7, 87.7)	78.1 (70.0, 93.0)	5.7
					
*All physicians*					
<5,000	233	54.9 (35.0, 90.4)	51.9 (32.0, 81.8)	64.9 (34.9, 96.6)	10.0
5,000–10,000	273	55.1 (36.4, 85.1)	54.9 (34.3, 84.9)	63.3 (36.9, 95.2)	8.2
10,000–30,000	509	85.6 (54.0, 135.7)	85.0 (52.8, 133.6)	93.4 (60.0, 139.3)	7.8
30,000–50,000	269	118.9 (82.0, 171.8)	119.6 (83.2, 169.0)	127.6 (88.4, 179.6)	8.6
50,000–100,000	301	131.5 (96.3, 171.7)	135.0 (96.3, 177.3)	144.9 (108.2, 187.7)	13.4
100,000–300,000	317	157.6 (120.7, 210.6)	162.2 (118.8, 222.3)	177.9 (135.4, 232.8)	20.3
≥300,000	71	171.1 (125.4, 222.4)	176.9 (132.0, 230.6)	198.3 (167.6, 312.0)	27.2

Municipal bodies are classified by the size of population, and the number of physicians per 100,000 population in each municipal body is shown by median (25-percentile, 75-percentile).

### Effect of distribution of medical interns on mal-distribution of physicians

To assess whether the distribution of medical interns affects the mal-distribution of physicians, measures of mal-distribution of physicians were calculated with medical interns excluded from calculation (Table 3). All three measures of mal-distribution improved when medical interns were excluded.

**Table 3:** Measures of mal-distribution obtained by excluding medical interns from calculation

	Gini coefficient	Atkinson index	Theil index
All physicians	0.3306	0.0956	0.2167
Physicians (excluding medical interns)	0.3177	0.0888	0.2007

Three measures of mal-distribution in 2006 were compared with measures of mal-distribution that were calculated with exclusion of medical interns from the number of physicians.

## Discussion

Although the number of physicians increased every year between 1996 and 2006, the overall ratio of physicians to population is still below the international standard [3]. In addition, the distribution of hospital physicians worsened during that period, especially after 2004. The number of hospital physicians increased in large urban areas but remained the same or decreased in rural areas, resulting into exacerbation of mal-distribution of physicians between urban and rural areas. This trend was not so obvious in the distribution of the physicians practicing at clinics. These results suggest that the doctor shortage problem in Japan is linked to both the shortage in absolute number of physicians and mal-distribution of hospital physicians.

There were several possible reasons for the mal-distribution of physicians working at hospitals. First, the decrease in the number of hospitals or hospital beds in Japan might have affected the distribution of physicians. The number of small-to-medium-sized hospitals, many of which are located in rural areas, has been decreasing over the past 30 years in Japan. The number of hospital beds has thus also been decreasing. For example, the number of acute care beds per 1,000 population was 11.8 in 1996 and decreased to 8.4 in 2004 and to 8.2 in 2006 [3]. The decrease in the number of the hospitals and number of hospital beds resulted in a shift of patients as well as physicians to the remaining large-sized hospitals, many of which are located in urban areas. Second, the introduction of the new postgraduate internship system has caused a concentration of new medical graduates or medical interns to urban areas. The new system requires medical school graduates to undergo clinical training for two years, and graduates can freely choose hospitals in which they want to work as medical interns [5-7]. Medical interns have shown a preference for general hospitals in urban areas rather than local university hospitals [1]. This tendency is reflected in the concentration of physicians to general hospitals located in urban areas (Table 2) and the very small increase in number of physicians working at university hospitals after 2004 (Figure 1). The manpower of medical interns as physicians and their concentration in urban areas cannot be ignored considering the large number (about 7,500) of medical graduates who start working as physicians at hospitals every year. In fact, when medical interns were excluded from calculation, all three measures of mal-distribution improved (Table 3). Since deterioration of the measures of mal-distribution occurred after 2004, it seems that the introduction of the new internship system has had a profound effect on the mal-distribution of physicians. Third, there is now no efficient system for correcting the imbalance in the distribution of physicians in urban and rural areas. Before the introduction of the new internship system, the majority of graduates began their carriers as residents at university hospitals. Professors of each specialty of university hospitals assigned positions in university hospitals or collaborating hospitals not only to medical interns but also to the other young physicians [1,8]. Hospitals located in remote and rural areas could recruit young physicians by this assignment. However, the university hospitals have now lost control over management of physicians' resources because of insufficient physicians' manpower. No alternative authorities to normalize the mal-distribution exist.

The outcome of the reform of the postgraduate internship system and its effect on physician distribution in Japan are still unclear, but deterioration in the quality of care provided by hospitals located in remote and rural areas due to insufficient manpower is unavoidable [17,18]. One possible way to prevent this problem is to increase the total number of physicians. In 2008, the Japanese government allowed an increase in new student quotas for medical schools, contrary to the long-standing governmental policy [19]. However, it is expected that a balance between physician supply and demand will not be achieved until 2022. Therefore, a shortage of doctors in remote and rural areas and a concentration of physicians in large urban areas will be the long-term trend in Japan. According to WHO's World Health Statistics 2007, Japan ranked as having the highest health status as indicated by healthy life expectancy at birth [3]. One of the main reasons for the excellent health status has been free access to healthcare services under the national insurance system covering all citizens in Japan [20,21]. The mal-distribution of hospital physicians might become a barrier that limits access to healthcare services in remote and rural areas, which might affect the health status of Japan's citizens. Further studies are needed to evaluate the mal-distribution of physicians and its effects on healthcare status in Japan.

One limitation of this study is that only physicians to population ratio was used for assessing mal-distribution of physicians. The ratio of physicians to population was not adjusted by health status, healthcare utilization or healthcare needs. Another limitation is that only data available for number of medical interns working in each municipal body were data for 2006. Therefore, the direct relationship between distribution of medical interns and mal-distribution of physicians can only be analyzed on a single year basis.

## Conclusion

The number of physicians in Japan increased every year between 1996 and 2006, but it is still below the international standard. In addition, the distribution of hospital physicians worsened during that period. The emerging problem of a doctor shortage in Japan is due to both a shortage in absolute number of physicians and mal-distribution of hospital physicians. The new postgraduate internship system might worsen this situation.

## Competing interests

The author declares that they have no competing interests.

## Authors' contributions

ST is solely responsible for this manuscript.

## References

[B1] Agence Francwe Presse. http://afp.google.com/article/ALeqM5i5XP-O252HC9opxHZ6aKgsXRKjqw.

[B2] The Japan Times. http://search.japantimes.co.jp/cgi-bin/ed20071001a1.html.

[B3] World Health Organization. http://www.who.int/whosis/whostat2007/en/index.html.

[B4] OECD OECD health data 2006.

[B5] Tsuchiya KJ, Takei N (2004). Focus on psychiatry in Japan. Br J Psychiatry.

[B6] Teo A (2007). The current state of medical education in Japan: a system under reform. Medical education.

[B7] Kozu T (2006). Medical education in Japan. Acad Med.

[B8] Ebihara S (2007). More doctors needed before boosting clinical research in Japan. Lancet.

[B9] Kobayashi Y, Takaki H (1992). Geographic distribution of physicians in Japan. Lancet.

[B10] Ministry of Health, Labour and Welfare of Japan. http://www.dbtk.mhlw.go.jp/IPPAN/ippan/scm_o_NinshouNyuuryoku.

[B11] Theodorakis PN, Mantzavinis GD (2005). Inequalities in the distribution of rural primary care physicians in two remote neighboring prefectures of Greece and Albania. Rural Remote Health.

[B12] Horev T, Pesis-Katz I, Mukamel DB (2004). Trends in geographic disparities in allocation of health care resources in the US. Health policy.

[B13] Morris S, Sutton M, Gravelle H (2005). Inequity and inequality in the use of health care in England: an empirical investigation. Social science & medicine.

[B14] Williams RF, Doessel DP (2006). Measuring inequality: tools and an illustration. International journal for equity in health.

[B15] Hann M, Gravelle H (2004). The maldistribution of general practitioners in England and Wales: 1974–2003. Br J Gen Pract.

[B16] Council of Local Authorities for International Relations. http://www.clair.or.jp/j/forum/series/pdf/fs09-en.pdf.

[B17] Pronovost PJ, Angus DC, Dorman T, Robinson KA, Dremsizov TT, Young TL (2002). Physician staffing patterns and clinical outcomes in critically ill patients: a systematic review. JAMA.

[B18] Kahn JM, Brake H, Steinberg KP (2007). Intensivist physician staffing and the process of care in academic medical centres. Quality & safety in health care.

[B19] Ministry of Eucation, Culture, Sports, Science and Technology of Japan. http://www.mhlw.go.jp/shingi/2008/06/dl/s0618-8a.pdf.

[B20] Toyabe S, Kouhei A (2006). Referral from secondary care and to aftercare in a tertiary care university hospital in Japan. BMC health services research.

[B21] Motomatsu K, Hirata N, Imamura T (2002). New trends in the Japanese medical system in 2002. Jpn Hosp.

